# Beyond Skin Deep: A Case Report of Infantile Systemic Hyalinosis in a Six-Month-Old Infant

**DOI:** 10.7759/cureus.59510

**Published:** 2024-05-02

**Authors:** Jayant D Vagha, Ajinkya Wazurkar, Bhushan Madke, Sham Lohiya, Shailesh Wandile, Keta Vagha, Chaitanya Kumar Javvaji, Suhit Naseri

**Affiliations:** 1 Pediatrics, Jawaharlal Nehru Medical College, Datta Meghe Institute of Higher Education and Research, Wardha, IND; 2 Dermatology, Venereology and Leprosy, Jawaharlal Nehru Medical College, Datta Meghe Institute of Higher Education and Research, Wardha, IND; 3 Pathology, Jawaharlal Nehru Medical College, Datta Meghe Institute of Higher Education and Research, Wardha, IND

**Keywords:** juvenile hyaline fibromatosis, antxr2 gene, hyaline, autosomal recessive, infantile systemic hyalinosis

## Abstract

A rare autosomal recessive condition called infantile systemic hyalinosis (ISH) is characterized by early-onset skin lesions that progress to the formation of numerous contractures. The underlying disease is the progressive accumulation of hyaline substances in many tissues. We are presenting the case of a male infant who was referred for evaluation and management at the age of six months. The infant had a history of recurrent episodes of diarrhea and showed limited movement in all four limbs. Upon physical examination, hyperpigmented papulonodular lesions on bony prominences and perianal regions were found, coupled with contractures in the elbow and knee joints. Hyaline deposition in the mid-dermal region was confirmed by histopathological analysis of a skin biopsy sample. The baby also had acute otitis media, which needed to be treated with antibiotics. Parents were counseled regarding the disease's diagnosis, complications, prognosis, and inheritance pattern. This case highlights the clinical presentation, diagnostic process, and management strategies employed in the care of ISH, emphasizing the importance of early recognition and multidisciplinary management in mitigating its devastating effects.

## Introduction

Infantile systemic hyalinosis (ISH) is an autosomal recessive disorder, often sporadic. It is characterized by skin nodules, recurrent infections, joint hyperpigmentation, joint stiffness, and gingival hyperplasia, typically presenting within the first few months of life or at birth [1]. The pathophysiology of ISH is the increasing deposition of hyaline material in multiple tissues [2,3]. The Anthrax toxin receptor 2 gene (ANTXR2) encodes the extracellular protein-binding domain of capillary morphogenesis protein-2 (CMG2). The site of the underlying genetic mutation causes ISH [4-6]. Due to the abnormal buildup of hyaline material caused by this mutation, juvenile hyaline fibromatosis (JHF), a milder form of ISH, is distinguished from it. Similar symptoms to JHF include painful joint contractures, perianal and subcutaneous fleshy nodules, and gingival enlargement [7]. Multiple contractures arise after early-onset skin lesions. Although histological evidence is frequently necessary, the primary basis for clinical ISH diagnosis is typical symptoms. Although medical knowledge has advanced, there is still no specific treatment for ISH; instead, patients’ quality of life is improved through physical therapy and nutritional care. Sadly, a large number of children with ISH pass away during the first two years of their lives, mainly as a result of severe diarrhea and recurring respiratory infections [3]. This case report highlights the case of an infant with an ISH diagnosis; we detail the clinical signs and symptoms, diagnostic process, and therapeutic strategies employed in the treatment of this rare and fatal condition. 

## Case presentation

A six-month-old male infant was referred to our tertiary care hospital from a medical camp due to concerns of minimal movement in all four limbs since the age of five months. The infant, born to non-consanguineous parents via normal vaginal delivery at term gestation with a birth weight of 2.8 kg, had experienced multiple admissions in the past five months for hypotonia and recurrent infections. Notably, the infant's mother observed decreased limb movements and the appearance of multiple black lesions on the skin starting at one month of age. Additionally, the child had a history of frequent episodes of diarrhea, necessitating treatment from a local hospital. Family history revealed no similar complaints among siblings.

**Figure 1:**
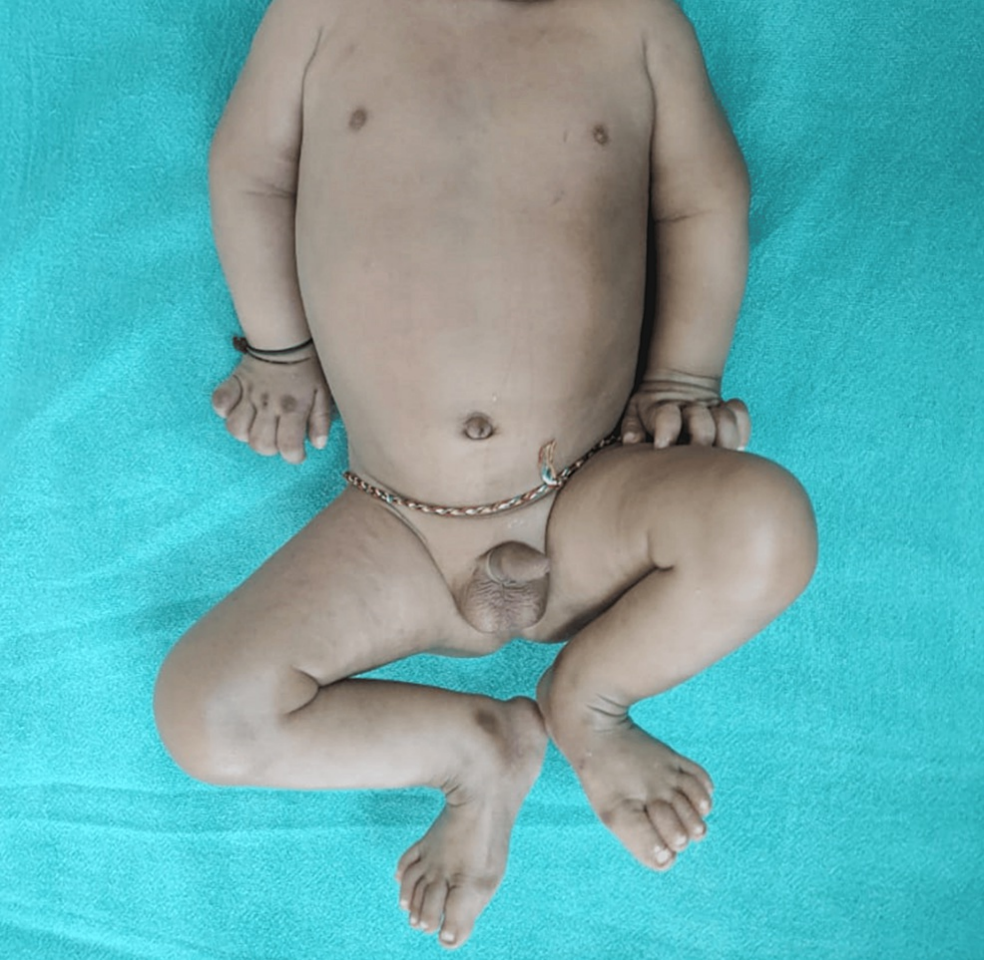
Multiple brownish papulonodular lesions on bony prominences with flexion contractures on the elbow and knee joints

**Figure 2:**
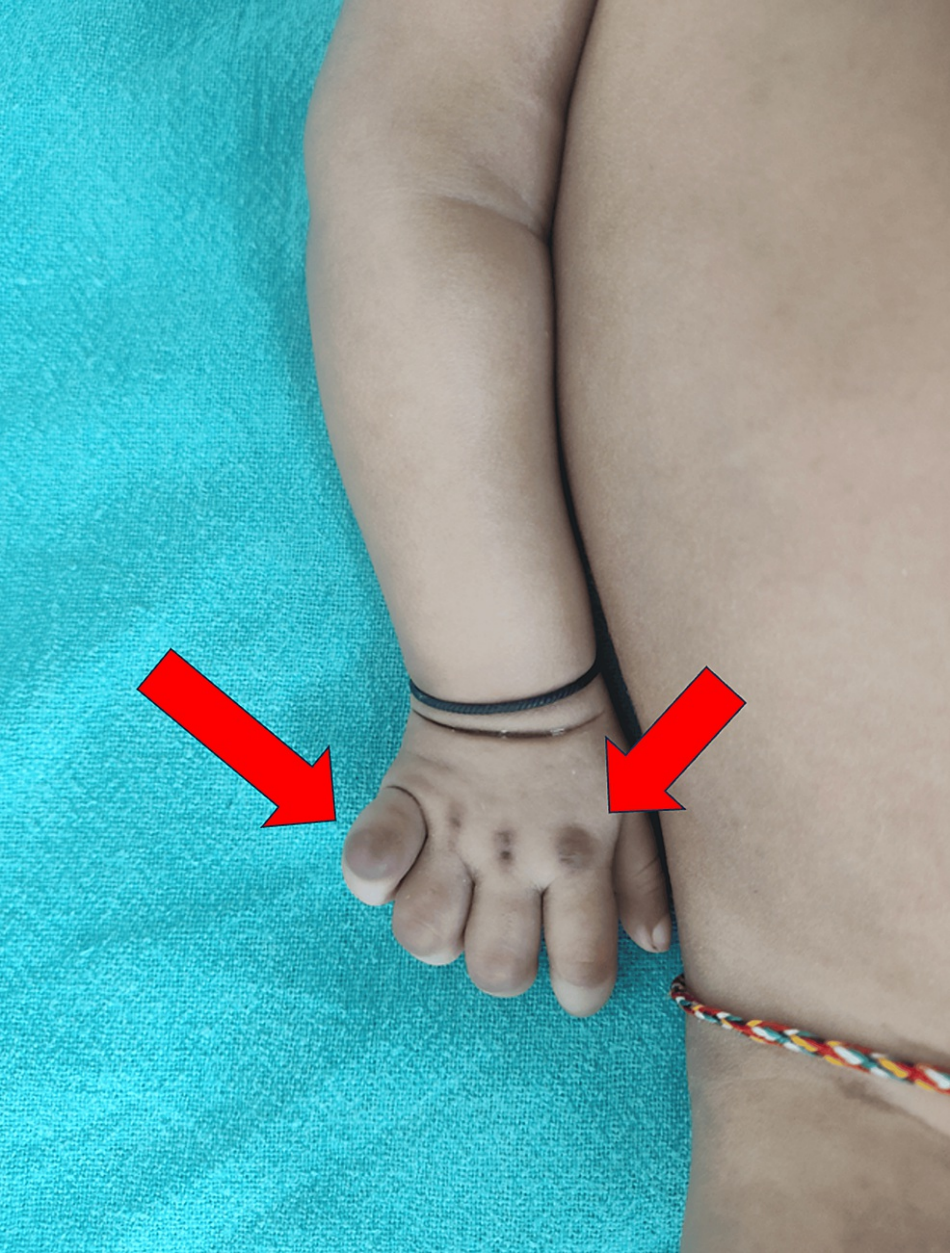
Hyperpigmented papulo-nodules on knuckles and metacarpophalangeal joints (red arrows)

The infant's vital signs were normal at the time of admission. A physical examination identified contractures in the knee and elbow joints, with the knee joints showing the most restricted range of motion. Hyperpigmented brownish papulonodular lesions were observed on bony prominences of the knuckles, metacarpophalangeal joints, and toes, accompanied by flexion contractures in the elbow and knee joints (Figures 1, 2).

**Figure 3:**
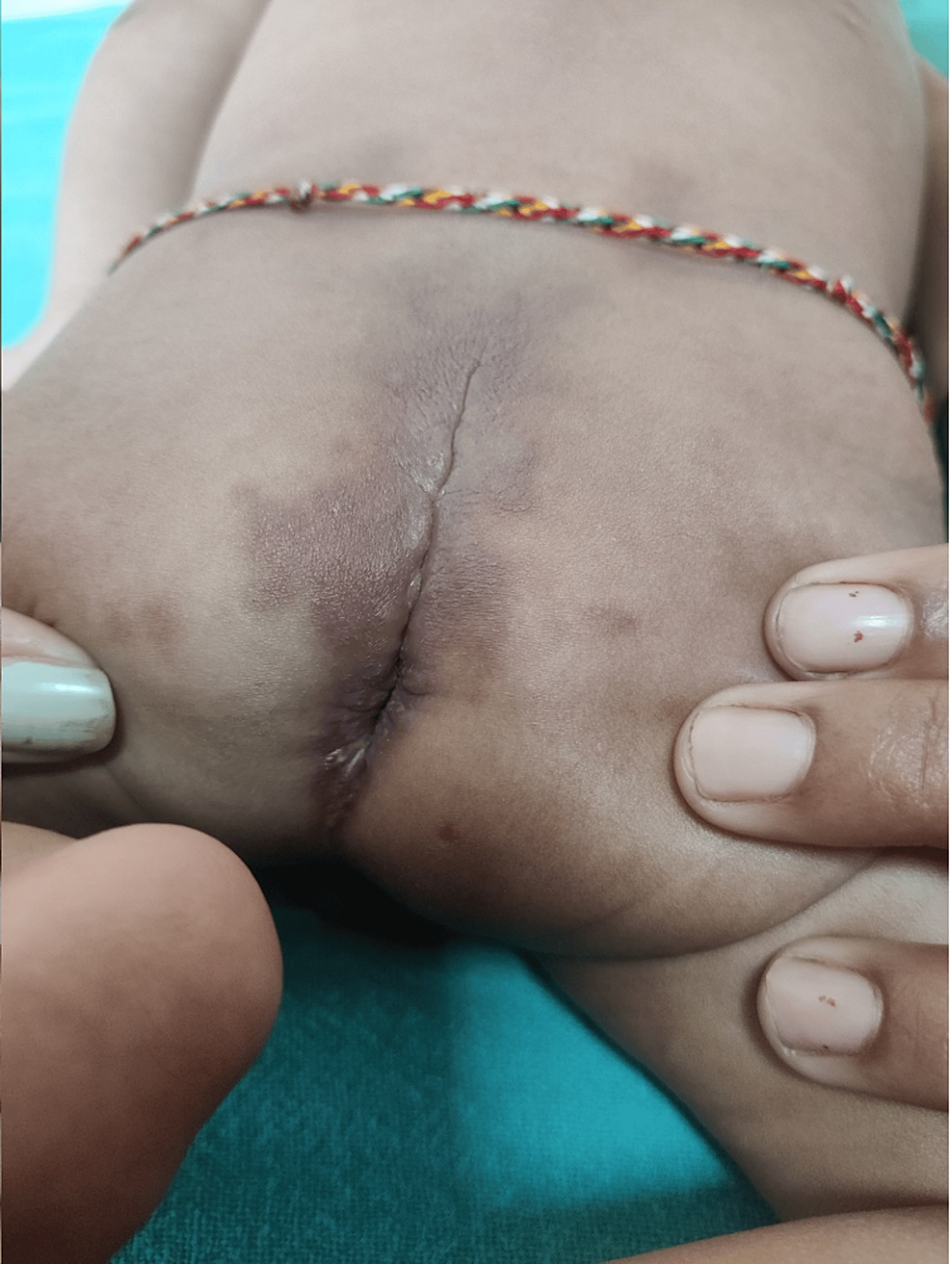
Hyperpigmented fleshy lesions were observed in the perianal area

Upon examination of the back, the infant exhibited dark brown papulonodular lesions over the sacral region and flexion contractures. Additionally, hyperpigmented fleshy lesions were observed in the perianal area, as depicted in Figure 3.

**Figure 4:**
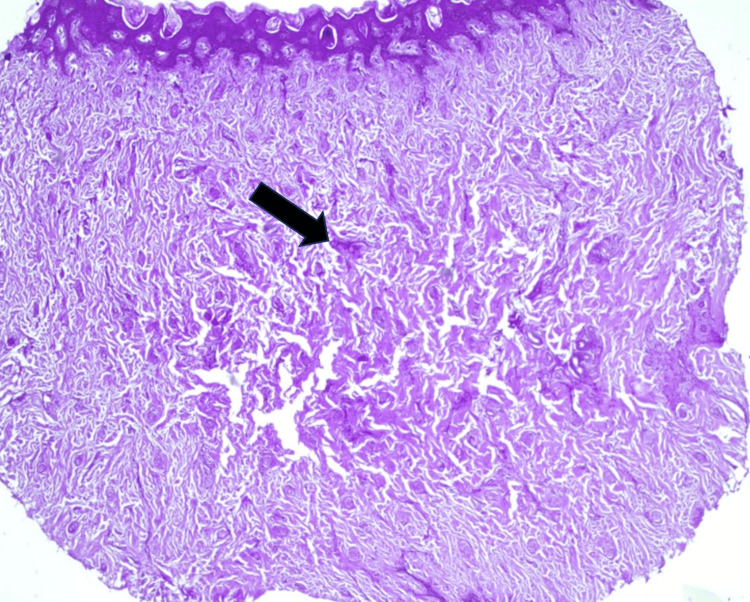
Histopathological examination shows periodic-acid Schiff hyaline deposition in the dermal region (black arrow)

Systemic examination yielded unremarkable findings, and laboratory investigations remained within normal ranges. The ophthalmological examination was also normal. Given the presence of skin lesions, a dermatology consultation was sought, resulting in a skin biopsy performed at the knuckle area. Histopathological examination revealed a reticulated epidermis with branching and fused rete ridges, mild or few keratotic hyperkeratosis, and papillomatosis. Notably, the dermis exhibited the deposition of hyaline material along with spindle cells arranged singly and in fascicles (Figure 4).

Furthermore, the infant presented with ear discharge, prompting an ear, nose, and throat (ENT) consultation, where acute otitis media (ASOM) was diagnosed and treated with antibiotics, leading to symptom resolution within 5-6 days. Parents were counseled regarding the disease's diagnosis, potential complications, prognosis, and inheritance pattern. Subsequently, the infant was discharged with instructions for follow-up after eight days. Unfortunately, despite our efforts, the child was lost to follow-up.

## Discussion

Infantile systemic hyalinosis (ISH) is a deadly autosomal recessive illness that causes hyaline deposition in numerous body tissues. Because of its inheritance, it is more common in consanguineous marriages [8]. Nezelof et al. initially reported ISH in a one-month-old Portuguese infant who had painful joint restriction, skin hypertrophy, and gingival hypertrophy in 1978 [9]. In 1986, Landing provided a comprehensive clinical description of ISH, elucidating the clinical characteristics of four infants of Mexican American descent who succumbed before reaching the age of 20 months [10].

Chromosome 4q21.21 is associated with hyalinosis, a disorder marked by an abnormal buildup of hyaline in different body tissues. ISH results from deletion mutations in the CMG2 (capillary morphogenesis protein gene 2) or ANTXR2 (anthrax toxin receptor 2) genes. The formation of capillaries and the integrity of basement membranes, which maintain and divide cells within tissues, depend on a protein encoded by the ANTXR2 gene. Defective production of glycosaminoglycans affects collagen production, and the CMG2 gene binds to type 4 collagen and laminins, essential to the basement membrane's strength. The production of basement membranes is disrupted by ANTXR2 mutations, which cause hyaline material to leak and accumulate in different organs, causing symptoms. ISH is inherited in an autosomal recessive manner. Affected individuals receive two mutant copies of the gene, one from each parent, and usually do not exhibit symptoms of the disorder [11].

The clinical presentations of Infantile Systemic Hyalinosis (ISH) and Juvenile Hyaline Fibromatosis (JHF) exhibit similarities due to defects in the anthrax toxin receptor 2 (ANTXR2) gene. Common clinical features include pain upon minimal handling, progressive joint contractures affecting small joints of both upper and lower limbs, gingival hypertrophy, and the presence of subcutaneous and perianal fleshy nodules, all of which share histological similarities. Distinguishing between ISH and JHF can be challenging due to their overlapping clinical features. Clinical features of ISH, JHF, and our case are shown in Table 1. However, ISH typically manifests early with severe and rapidly progressive symptoms, often leading to fatality within the first two years of life and the possibility of developing malabsorption. Both conditions require a high level of clinical suspicion for an accurate diagnosis [12]. 

**Table 1:** Clinical features of ISH, JHF and our case ISH: Infantile Systemic Hyalinosis, JHF: Juvenile Hyaline Fibromatosis, Present (+), Absent (-)

Clinical features	ISH	JHF	Our case
Age of onset	Within first few months	< 5 years	1 month
Papular skin lesions	+	+	+
Thickened skin	+	-	+
Perianal nodules	+	+	+
Large nodules/ tumors	+	-	-
Hyperpigmented plaques	+	+	+
Joint contractures	+	+	+
Osteopenia	+	+	-
Pesistent diarrhea	+	-	+
Recurrent infections	+	-	+
Visceral involvement	+	-	-
Prolonged survival	-	+	-

ISH can be diagnosed prenatally by chorionic Villus sampling when one diagnosis is established in the family already [13]. Joint contractures of both upper and lower limbs and smaller joints occur due to hyaline deposition on the skin and muscles [12]. There is deposition of collagen VI in the skin and connective tissue. The differential diagnosis of ISH involves juvenile hyaline fibromatosis, infantile stiff skin syndrome, congenital facial dystrophy, and Winchester syndrome. Infantile systemic hyalinosis and juvenile hyaline fibromatosis combine to form a spectrum called hyalinosis fibromatosis syndromes [14]. Patients usually die before three years of age, the causative factor being recurrent chest infections due to difficulty in chest wall movements [15].

ISH is an exceedingly debilitating illness for which an effective treatment remains elusive. Ongoing studies are still underway to uncover potential solutions. While some cases have shown marginal improvement in joint movement and flexibility with oral d-penicillamine, it's only a minority that benefits from this intervention. The primary strategies for enhancing the quality of life in affected individuals include physiotherapy, nutritional support, and pain management, though these measures only provide partial relief. Accurate diagnosis of ISH and genetic analysis can empower families to avoid unnecessary and distressing procedures for their children and mitigate the risk of recurrence within their lineage [16].

## Conclusions

In conclusion, we presented a case of infantile systemic hyalinosis (ISH) in an infant with characteristic clinical features, including early-onset skin lesions, multiple contractures, and recurrent infections. Histopathological examination confirmed the presence of hyaline material deposition in the dermis. Prompt multidisciplinary management, including dermatology and otolaryngology consultations, was essential in addressing associated complications. This case underscores the importance of early recognition, comprehensive evaluation, and coordinated multidisciplinary care in optimizing outcomes for patients with ISH. Further research and awareness are warranted to enhance understanding and management of this rare and debilitating condition.
